# Complete genome sequence of *Streptococcus suis* isolated from an aborted bovine fetus and placenta in British Columbia, Canada

**DOI:** 10.1128/mra.01342-24

**Published:** 2025-05-22

**Authors:** Kazal Ghosh, Anatoliy Trokhymchuk, Stephen Raverty

**Affiliations:** 1 Animal Health Centre, Ministry of Agriculture and Food, Government of British Columbia 102795, Abbotsford, British Columbia, Canada; 2 Department of Large Animal Clinical Sciences, Western College of Veterinary Medicine, University of Saskatchewan 70399, Saskatoon, Saskatchewan, Canada; University of Maryland School of Medicine, Baltimore, Maryland, USA

**Keywords:** complete genome, *Streptococcus suis*, cattle, abortion, British Columbia, Canada

## Abstract

We present the complete genome of *Streptococcus suis* KKAHC02 isolated from an aborted bovine fetus and placenta in British Columbia, Canada. The genome consists of a circular chromosome of 2,340,488 bp long and five circular plasmids of 5,796, 4,273, 3,973, 3,964, and 2,832 bp, respectively.

## ANNOUNCEMENT

*Streptococcus suis* is a recognized pathogen of swine with clinical manifestations, including meningitis, septicemia, and endocarditis (1). While its impact on pigs is well documented, its role in other species, especially cattle and humans, is less well understood but is gaining prominence due to its zoonotic potential (2–4). Herein, we present the complete genome of an *S. suis* isolate recovered in 2021 from the fetus and placenta aborted by a 5-year-old cow in the province of British Columbia (BC).

**Fig 1:**
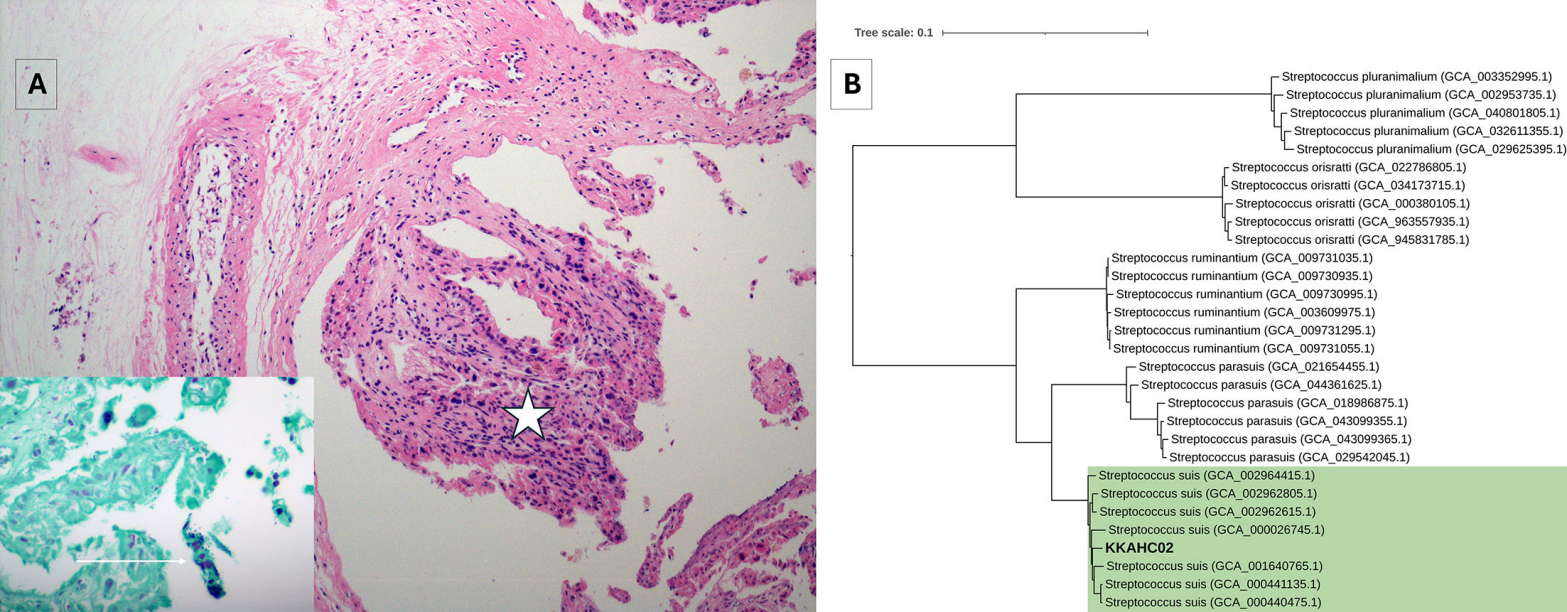
(A) Placentitis with expansion of chorioallantoic villi (star). The inset is a Gram stain; there are numerous gram-positive cocci and diplococci (arrow) with scattered gram-negative bacilli. (B) Midpoint-rooted maximum likelihood phylogenetic tree of *S. suis* KKAHC02 and some selected *S. suis*, *S. suis*-like, and *Streptococcus pluranimalium* genomes obtained from NCBI GenBank. The tree was obtained with GToTree using the Firmicutes predefined gene set of 119 single-copy genes and default parameters, and the tree was viewed by iTOL.

Histopathology revealed multifocal placentitis with numerous intralesional gram-positive cocci (Fig. 1A). Tissues submitted for routine bacteriology were plated onto Blood and MacConkey agar (Oxoid) and incubated aerobically at 35°C with 5% CO_2_ for 18–24 hours. Colonies were identified by MALDI-TOF MS (Bruker). Heavy growth of *S. suis* with moderate *Escherichia coli* was recovered from the placenta and fetal lung. The histopathology and microbiology findings suggested *S. suis-*associated abortion. To our knowledge, this is one of the first reported cases of *S. suis* in BC’s dairy cattle.

Bacterial DNA was extracted from pure colonies using the MasterPure Complete DNA and RNA Purification Kit (LGC) for Oxford Nanopore Technologies (ONT) sequencing and the Qiagen DNeasy Blood and Tissue Kit (Qiagen) for Illumina sequencing according to the respective manufacturer’s instructions. DNA concentration and purity were measured with a NanoDrop spectrophotometer (Thermo Fisher Scientific) and quantified with a Qubit 1× dsDNA BR Assay. Nanopore DNA library was prepared with a Native Barcoding Kit (Oxford Nanopore Technologies), loaded onto a flow cell (version 9.4.1), and sequenced for up to 12 hours on a GridION Mk1. ONT reads were basecalled using Guppy (version 5.0). Illumina library preparation followed the DNA Preparation Kit protocol. PhiX control was spiked in at 2% for quality control, and sequencing was performed using the MiniSeq High Output Reagent Kit, with 318 cycles of paired-end 150 bp reads on the Illumina MiniSeq System.

Nanopore reads were demultiplexed, and adaptors were removed using Porechop (version 0.2.4). Sequencing quality was assessed with NanoPlot (version 1.42.0) (5), and low-quality reads were filtered using Chopper (version 0.7.0) (5), selecting reads with a minimum length of 2,000 bp and a *q*-score of 30. Illumina reads were quality checked using FastQC (version 0.12.1). The KKAHC02 genome was hybrid-assembled with both Nanopore and Illumina reads, circularized, and rotated to DnaA using Unicycler (version 0.5.1) (6). Genome quality was assessed with Quast (version 5.2.0) (7), and completeness was checked using BUSCO (version 5.6.1) (8). Genome annotation was done using PGAP (version 6.9) (9). Chromosomes and contigs matching a plasmid were detected using PlasFlow (10). Default parameters were used except where otherwise noted.

The assembled genome consists of a complete single circular chromosome and five circular plasmids (Table 1). BUSCO analysis indicated 98.4% genome completeness. GC content and annotation details are provided in Table 1. Furthermore, we constructed a phylogenetic tree of KKAHC02 and some selected *S. suis, S. suis*-like (*Streptococcus ruminantium*, *Streptococcus orisratti*, and *Streptococcus parasuis*), and *S. pluranimalium* genomes with GToTree (11) using the Firmicutes predefined gene set of 119 single-copy genes. The isolate from this study clustered with *S. suis* genomes (Fig. 1B).

**TABLE 1:** Sequencing statistics, genome, and annotation details of *Streptococcus suis* strain KKAHC02

Parameters	Genomic features
Genome	Size (bp) and GenBank accession number
Chromosome	2,340,488 (CP176418)
Plasmids	
pKKAHC01	5,796 (CP176419)
pKKAHC02	4,273 (CP176420)
pKKAHC03	3,973 (CP176421)
pKKAHC04	3,964 (CP176422)
pKKAHC05	2,832 (CP176423)
GC content (%)	41.27
Illumina statistics	
Read length	149
Number of reads (paired, 2×)	837,511
Mean depth	53×
ONT statistics	
*N*_50_ (bp)	5,637
Mean read length (bp)	3,182
Number of reads	91,297
Total bases	290,575,824
Coverage	126×
Annotation details	
CDSs	2,285
rRNAs	12
tRNAs	57
ncRNAs	4
Pseudogenes	100
CRISPR arrays	1

## Data Availability

Assembled genome was deposited in the NCBI GenBank under the accession numbers listed in Table 1. The sequencing reads are stored in the Sequence Read Archive under the numbers SRX27073687 (Nanopore) and SRX27073686 (Illumina). All data are encompassed under BioProject number PRJNA1198093.
